# Multiphysics Measurement Method for Supercapacitors State of Health Determination

**DOI:** 10.3390/mi16111295

**Published:** 2025-11-19

**Authors:** Thomas Doucet, Jean-François Mogniotte, Raphaël Amiot, Alaa Hijazi, Pascal Venet, Minh-Quyen Le, Pierre-Jean Cottinet

**Affiliations:** 1 Université Claude Bernard Lyon 1, INSA Lyon, Ecole Centrale de Lyon, CNRS, Ampère, UMR5005, F-69100 Villeurbanne, France; alaa.hijazi@insa-lyon.fr (A.H.); pascal.venet@univ-lyon1.fr (P.V.); 2 Univ Lyon, INSA-Lyon, LGEF, UR682, F-69621 Villeurbanne, France; jean-francois.mogniotte@insa-lyon.fr (J.-F.M.); minh-quyen.le@insa-lyon.fr (M.-Q.L.)

**Keywords:** supercapacitor, gauge strain measurement, ageing characterizations, mechanical deformation, analytical modelling, state of health

## Abstract

This work presents a comparative study on the ageing of supercapacitors and a method for monitoring their state of health (SoH) through mechanical deformation. This study aims to evaluate the accelerated ageing behaviours of these systems under specific cycling conditions and temperatures, allowing the establishment of a correlation between SoH and casing deformation in supercapacitors. Experimental ageing tests revealed supercapacitors displayed an initial “burning” phase followed by a linear ageing trend. Strain gauges were employed to measure the mechanical deformation of supercapacitor casings, providing real-time insights into their SoH. Capacitance fading in supercapacitors was modelled using Brunauer–Emmett–Teller (BET) theory, hypothesizing that gas adsorption during ageing significantly contributes to performance decline. Model predictions were validated against experimental data, demonstrating a clear correlation between capacitance fading, internal resistance, remaining energy, and casing deformation. This work highlights the potential of mechanical deformation monitoring as a practical and non-invasive approach for assessing the SoH of supercapacitors.

## 1. Introduction

Climate change and the need to reduce greenhouse gas emissions have driven a growing interest in wind turbines, as well as electric vehicles and hybrid electric vehicles. The development and adoption of these technologies depend on the efficiency and durability of their energy storage systems, primarily Li-ion batteries and supercapacitors (SCs) [1,2]. Understanding the ageing behaviour of these storage systems is essential for improving performance and ensuring reliable maintenance. While Li-ion batteries remain the preferred choice for applications requiring high energy density [3,4,5], SCs are increasingly employed in high-power applications due to their superior power capabilities [6], despite the shorter lifetime and higher environmental impact of Li-ion batteries.

This increase in the use of SCs leads us to pay more attention to their End of Life (EoL) and more generally their State of Health (SoH) [7]. These two indicators are crucial to the development of a reliable monitoring tool for SCs. Accurate knowledge of an energy storage device’s SoH and its likely evolution is essential not only for predictive maintenance but also for optimizing balancing circuits to extend operational lifetime [8]. Usually, SoH online identification is based on observer or their balancing circuit [9,10] to produce a useful signal image of their lifetime.

For SCs, the primary SoH indicator is capacitance, while internal resistance is also a critical parameter, as it tends to increase with ageing. Conventionally, a 20% reduction in capacitance and a 200% increase in internal resistance are considered thresholds for reaching EoL, beyond which capacitance drops sharply, making SoH prediction particularly relevant. Current monitoring approaches are often invasive, potentially altering system operation. These approaches typically rely on either direct measurement of current and voltage or electrochemical impedance spectroscopy (EIS) [11,12,13]. While effective, these methods can be restrictive and intrusive [14,15,16,17], motivating the development of alternative strategies [18,19].

Indeed, mechanical and thermal characteristics are prone to change during ageing [8,20], due to the inherent volume change or inhomogeneities of ageing [21]. Then it is possible to link ageing with those characteristics changes to produce a faithful image of the current SoH. The method that we propose consists of monitoring the lifetime of SCs by implementing a multi physics sensor in the case of SCs. The sensor was placed at the location where mechanical deformation induced by various ageing mechanisms is expected to be most pronounced—namely, the degassing zone provided by the design of SCs [22]. In our case, this corresponds to the top of the structure. However, this localization could be adapted depending on the form factor and architecture of the devices under study [15,23,24]. The goal here is to provide both a case study and a general instrumentation methodology for the identification of multiphysical markers for the health monitoring of energy storage elements. This method allows direct implementation of sensors via an industrial process which gives an image of the SoH of the cell.

The main innovation of this work lies in demonstrating that mechanical deformation analysis can serve as an effective, non-invasive method to monitor SoH of commercial SCs. This approach builds on extensive experience with this technology, which is widely used in the context of Structural Health Monitoring (SHM) of aeronautical structures [25]. It also leverages the favourable transduction properties of strain gauges for monitoring the external casing of components [26,27,28,29,30]. This approach facilitates the development of a non-intrusive diagnostic and prognostic strategy that does not alter the internal structure of the devices. By combining detailed ageing characterization, tracking capacitance fading and resistance growth with strain gauge measurements of casing deformation, the study establishes a direct correlation between internal gas emission, mechanical expansion, and electrochemical performance. This approach provides a framework for real-time SoH assessment without intrusive testing. Moreover, the work opens avenues for future improvements, such as using rosette strain gauges for enhanced accuracy or flexible PCB sensors for better integration. Such a non-invasive monitoring strategy could be extended to other energy storage systems.

This article is organized as follows: Section 2 introduces the selected SCs, describes the test bench along with the cycling profile, and details the sensors design for the multiphysics measurement. Section 3 focuses on analyzing the correlation between material properties (electrical, mechanical deformation, and morphological characteristics) and the ageing process under different temperature, while also providing a discussion of the results. Finally, Section 4 highlights the key findings of this work and offers suggestions for future research and development.

## 2. Materials and Methods

### 2.1. Materials Selection

The SCs (ref. XV3560-2R7407-R) purchased from Eaton Corporation (KLN, Hong Kong, China) (see Table 1) were selected. It consists of a cylindrical cell architecture with carbon-based electrodes and an organic electrolyte. Their geometry allows them to canalize the deformation on the SCs top and thus to give a reliable measurement of the strain. Based on datasheet and manufacturer information, the active material of the electrodes consists of high-surface-area activated carbon deposited on aluminum current collectors. The electrolyte contains tetraethylammonium tetrafluoroborate (TEA-BF_4_) dissolved in acetonitrile (CH_3_CN), a commonly used organic solvent in commercial supercapacitors due to its high dielectric constant and excellent ability to dissolve a broad range of electrolyte salts [31,32].

The separator is typically composed of cellulose or polypropylene, ensuring ionic conductivity while preventing electrical short circuits [31]. The aluminum casing includes a degassing zone at the top to accommodate internal pressure build-up during cell ageing. This architecture also facilitates evacuation of excess gas such as in case of explosion; this part is deformed by the pressure increase inside the casing.

**Figure 1:**
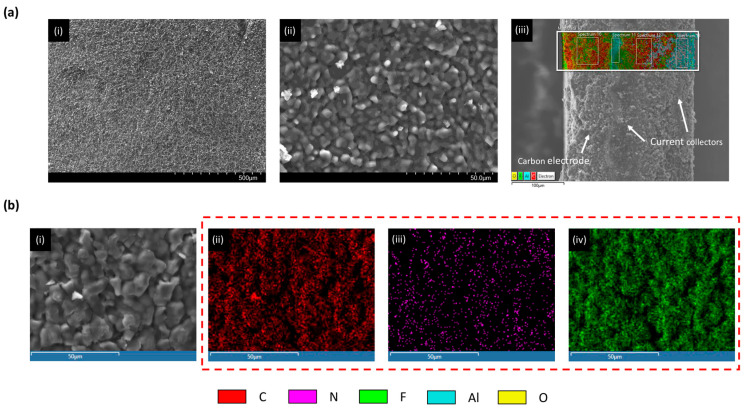
(**a**) (i,ii) SEM micrographs of a fresh activated carbon electrode acquired at different magnifications; (iii) SEM imaging combined with XDS analysis of carbon electrode slice showing the current collectors inside the electrode. (**b**) (i) High-magnification SEM image of the carbon electrode surface; (ii–iv) XDS spectra of the same region, indicating the presence of carbon (C), nitrogen (N), and fluorine (F).

The chemical composition of the supercapacitor was further confirmed through scanning electron microscopy (SEM) and energy-dispersive X-ray spectroscopy (XDS). A fresh electrode sample, obtained from a supercapacitor discharged to 0% state of charge (SoC) and opened inside a glove box under controlled conditions, was used for analysis. SEM imaging revealed a porous carbon structure typical of activated carbon electrodes (Figure 1a(i–iii),b(i)). XDS analysis identified the presence of fluorine, thereby confirming the use of TEA-BF_4_ as the conducting salt in the acetonitrile-based electrolyte (Figure 1a(i),b(i–iv)). These findings validate the assumed composition and provide direct microstructural and elemental evidence of the materials involved in the electrochemical system. SEM images were acquired with secondary electron configuration using a 15 keV electron beam.

This composition is representative of commercial EDLC (Electric Double Layer Capacitor) technology and is prone to gas generation under thermal and electrochemical stress, particularly involving acetonitrile decomposition and carbon surface reactions.

### 2.2. Methods of Characterization

#### 2.2.1. Ageing Setup

**Figure 2:**
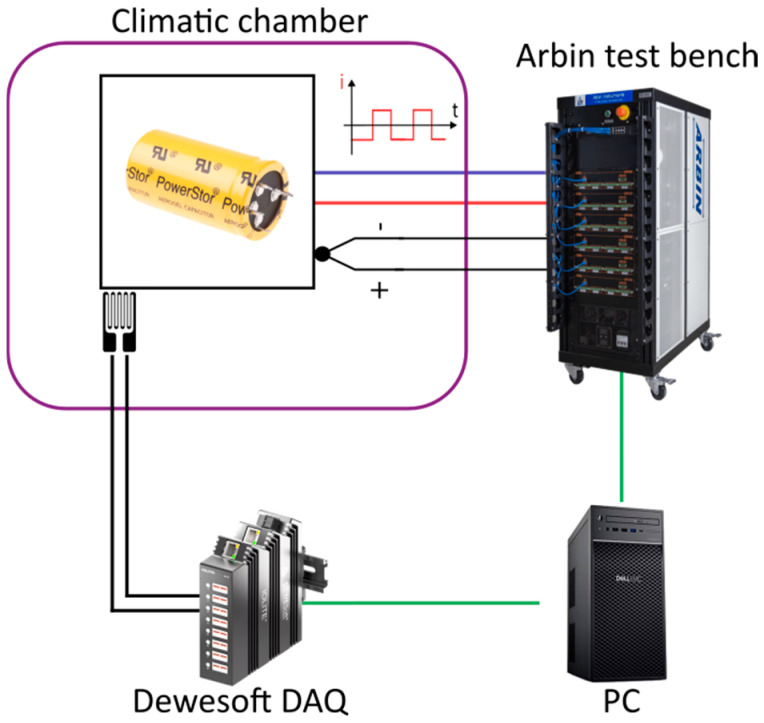
Test bench overview.

The test bench consists of an Arbin BT2020 10 A 5 V with multi-channel charge/discharge testing system (Arbin Europe, Munich, Germany); two IOLITE 8STGS data acquisition systems (DAQs) (DEWESoft France, Massy, France) for strain measurements; and two climatic chambers: a XUII2 France oven and a Memmert model 500 (Memmert, Schwabach, Germany) for the tests at 50 °C and 60 °C, respectively (Figure 2). This setup provided a stable and repeatable framework to evaluate the lifetime and performance degradation of the tested SCs.

The SCs were subjected to cycling tests with a constant current. Three key parameters were recorded for each tested element including terminal voltage, casing temperature, and mechanical strain at the top of the casing. Each cell was connected to a terminal board with four connections: two for the current cycling supply from the Arbin test bench and the two others for measurement purposes.

To accelerate the ageing process, thermal ageing tests were conducted at 50 °C and 60 °C. Elevated temperatures accelerate chemical and physical degradation, allowing key ageing mechanisms to be observed within a shorter experimental timeframe [34]. The chosen temperatures were dictated by the operational limits and stability of the climatic chambers, ensuring reproducible and controlled testing conditions.

**Figure 3:**
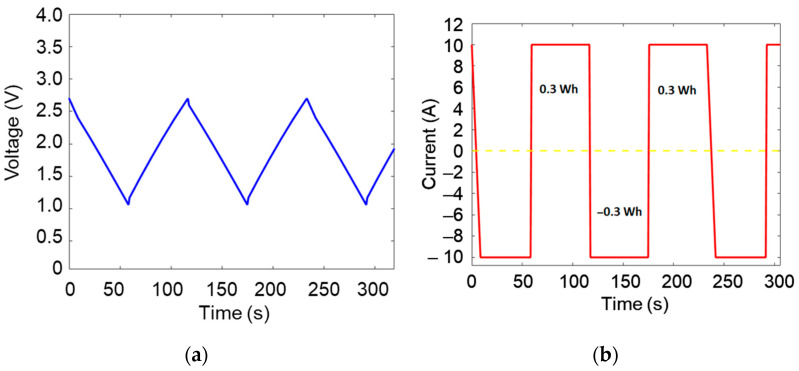
(**a**) Voltage and (**b**) current profiles of the galvanostatic cycles.

Eight samples were conducted at 10 A (the maximum of the Arbin cycler). Each set of samples was operated at two different temperatures: 50 °C and 60 °C. Ageing was carried out through cycling with microcycles, during which a portion of the total energy of the studied storage systems was repeatedly charged and discharged. The cycling energy was limited to 0.3 Wh per charge/discharge microcycle (Figure 3a,b), corresponding to a voltage range between 2.7 V (V_r_) and 1.35 V (0.5 V_r_) for a fresh SC. After a 5-day cycling, voltage-current characterization was conducted on each sample. The capacitance was determined based on the methodology described in the IEC 62391 standard [35,36].

**Figure 4:**
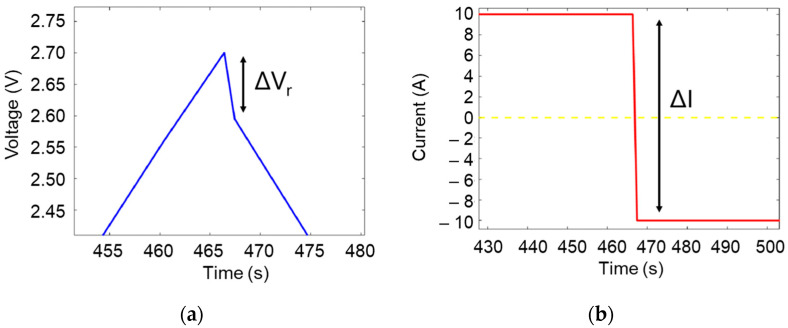
(**a**) Voltage and (**b**) current profiles associated with the ohmic voltage drop.

The equivalent series resistance (ESR) can be determined thanks to instantaneous voltage drop ( $\Delta V_{R}$) and the current change ( ΔI) between two cycles (see Figure 4a,b).

#### 2.2.2. Strain Measurement

**Figure 5:**
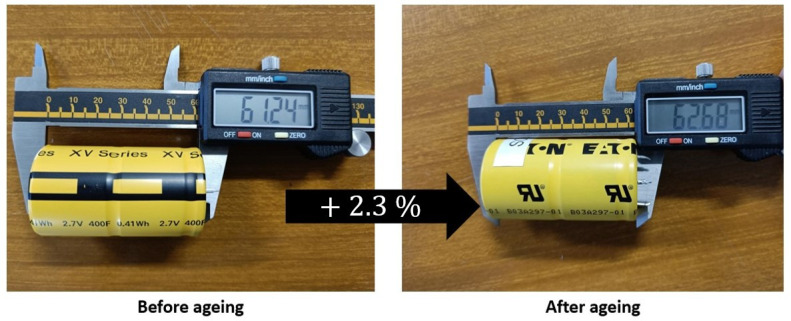
Strain measurement of a SC casing before and after the ageing process.

In order to choose relevant physical parameters to be monitored, the dimensions of SC are first observed before and after ageing cycling [6]. As indicated in Figure 5, inflation located on the top side of the battery confirms an increase in its height of ~2.3%. This non-negligible increase can be measured by monitoring the strain evolution of the casing during the SCs lifecycles. Major disturbance factors like thermal stress and/or internal pressure changes, which might affect the stress measurement, should be considered. Based on the current understanding of the SC ageing process, gas emissions appear to be strongly linked to a decrease in the performance of these systems [8].

To monitor mechanical deformation of the SC, Daokai BF120-3AA (Daokai, Guangzhou, Hong Kong) strain gauges (with a gauge factor of k = 2.1, and a measurable range of 20,000 µdef) were used in this study to attach with ethyl cyanoacrylate super glue. The area of the wire grid was 3.0 × 2.44 mm^2^. A sampling frequency of 20 Hz was chosen for data acquisition and recording, which was considered fast enough relative to the dynamics of the system.

The relative change in resistance of the gauge (denoted ΔR/R) was recorded and then converted into micro-strain (denoted µ-strain, unit in µdef) via the DAQs according to the following equation:

(1) $$µ-\mathrm{strain}=\frac{\Delta R}{R}\frac{1}{k}$$

**Figure 6:**
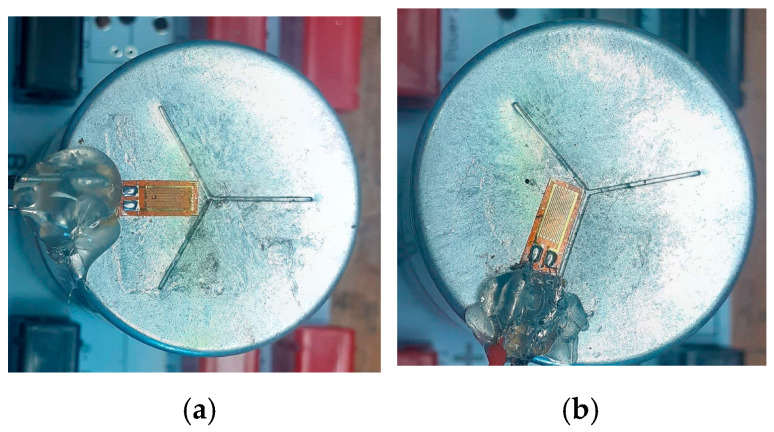
(**a**) The first and (**b**) the second implementation of the strain gauges on SCs.

Preliminary studies were conducted to determine which part of the SC was the most relevant for measurement with the strain gauge. Ultimately, the top of the casing, where deformation consistently increases, was chosen for monitoring throughout the entire ageing process. The strain gauges were attached to the top casing of the SCs, as shown in Figure 6a,b. They were placed in two different configurations to assess the influence of their placement on the measurement results.

**Figure 7:**
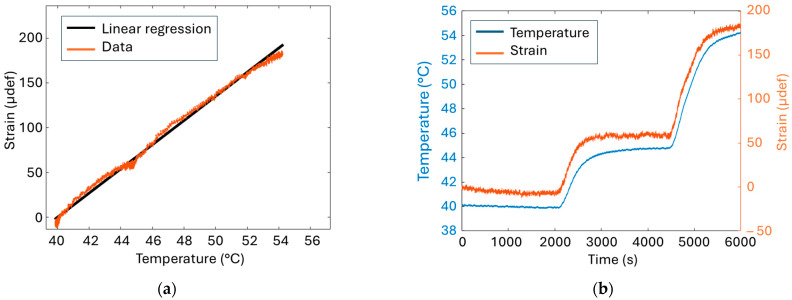
(**a**) Strain deformation versus temperature, (**b**) Temperature stability of the strain gauge when exposed to temperature variation over time.

From the practical point of view, temperature changes caused by Joule heating might somehow affect the strain measurement. To verify whether or not the strain gauge is stable during the temperature ageing process, the SC casing implemented with the gauge was placed in a climatic chamber where the set temperature was controlled. A relationship between the measured strain and the set temperature was established as displayed in Figure 7a. The coefficient heat output was empirically found equal to 13.5 µdef/°C, i.e., much higher than the one indicated in the datasheet of the strain gauge (2 µdef/°C). However, as illustrated in Figure 7b, the strain value remains stable and faithfully follows the temperature change, from 40 to 55 °C, which is roughly the working temperature of our test. Within this temperature range, a quasi-linear relationship between the strain and the temperature is observed, confirming the well-known behaviour of the strain gauge given by Equation (1).

**Figure 8:**
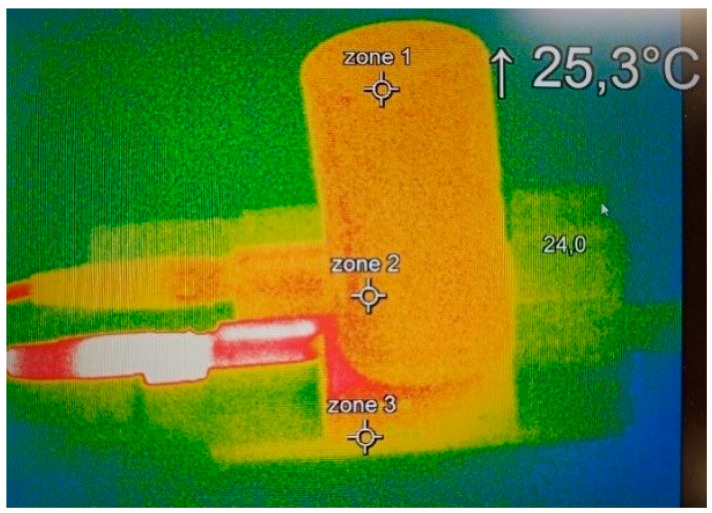
Thermal images of the SC.

Therefore, in this context, strain measurement appears to be a reliable indicator for assessing the SoH of SCs. It provides a direct way to quantify the amount of emitted gas, as the gas pressure deforms the casing. This deformation induces strain, which is then directly related to the ageing process. The results of Figure 7 clearly demonstrate that the value measured by the strain gauge is influenced by the thermal variations. Thermocouples have been thus implemented on the SCs to provide local temperature measurement. This type of measure is sufficient to monitor the temperature of the component, as confirmed by the thermal image camera shown in Figure 8. Indeed, these images reveal a homogeneous temperature distribution across the entire surface of both SC’s casing.

## 3. Results and Discussions

### 3.1. Ageing of Electric Properties

**Figure 9:**
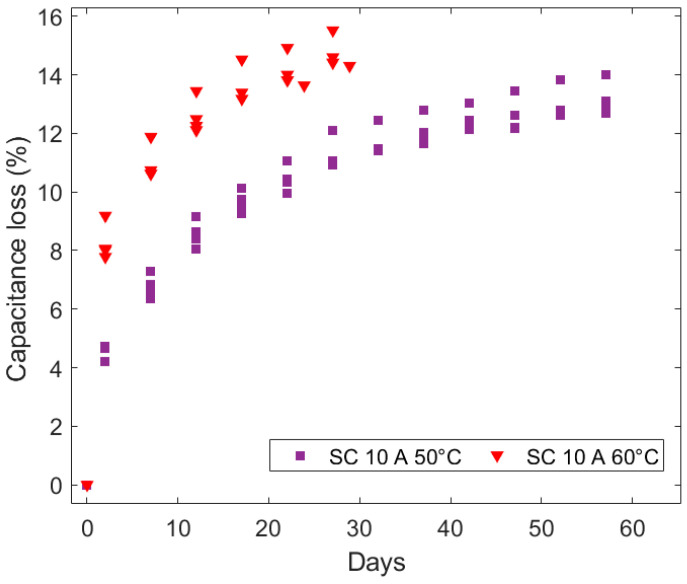
Capacitance loss (in percentage) versus the ageing duration (number of days).

Figure 9 illustrates the capacitance loss for various ageing tests of SCs under different temperature conditions, as a function of time (days). Regardless of the testing conditions, all datasets exhibit a progressive decrease in capacitance over time. The degradation rate remains consistent for a given temperature but differs between 50 °C and 60 °C, with some conditions experiencing faster loss than others. Over time, the rate of capacitance loss gradually decreases and tends to stabilize at a plateau of approximately 16% after about 30 days of ageing. The burn-in phase appears to be temperature-dependent, with rapid ageing diminishing after approximately 20 days (i.e., 15,000 cycles), as displayed in Figure 9.

To maintain stable operating conditions throughout the long-term cycling tests, the experiments were conducted up to approximately 16% capacitance reduction. This value, slightly below the 20% threshold used to define significant degradation, was chosen to prevent excessive ageing. The approach ensured that the cells remained in good functional condition during prolonged testing, while still allowing the observation of the initial degradation behaviour and strain evolution of the cell.

**Figure 10:**
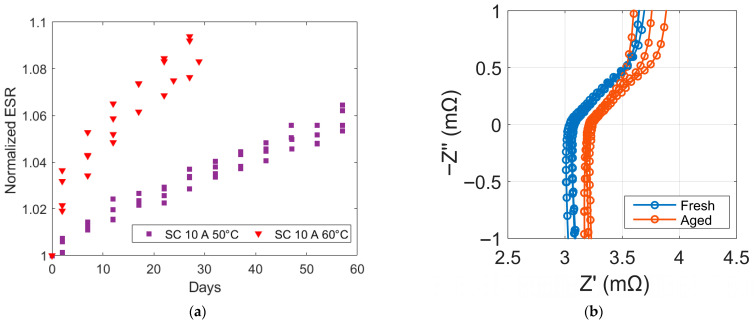
(**a**) Normalized resistance of SCs as a function of time. (**b**) Nyquist plots of the SCs before (Fresh) and after ageing (Aged) obtained by EIS at 5 A over the frequency range of 10 mHz and 10 kHz at 50 °C and 50% of SoC (i.e., 1.35 V).

As the capacitance decreases, the ESR within the SCs. The increase in ESR is a key indicator of the ageing process, as it significantly contributes to additional heat generation in the cell through Joule heating [37]. The contribution of entropy variation to heat generation in SCs is negligible, as charge storage is mostly electrostatic and the processes are largely reversible and isothermal. The evolution of normalized resistance in SCs is illustrated in Figure 10a.

The electrochemical impedance spectroscopy (EIS) measurements before and after ageing for the SCs tested at 50 °C are shown in Figure 10b as Nyquist plots. In the mid-frequency region, the real part of the impedance (Z’) is somewhat higher in the aged sample, indicating a higher series/internal resistance (ESR) [38]. The aged sample also exhibits a slightly larger arc and a slight reduced −Z″ peak magnitude, consistent with an increase in charge-transfer resistance and a reduction in the capacitive/ideal interfacial response. At low frequencies, the aged sample’s tail deviates from the near-vertical behaviour observed in the fresh sample, suggesting increased diffusion limitations or partial pore occlusion after ageing. The negative values of −Z’, particularly at high frequencies, reflects inductive behaviour arising from imperfect wiring, and also from the cylindrical winding of the electrodes [39].

### 3.2. Analytical Modelling Based on Strain and Pressure Measurements

**Figure 11:**
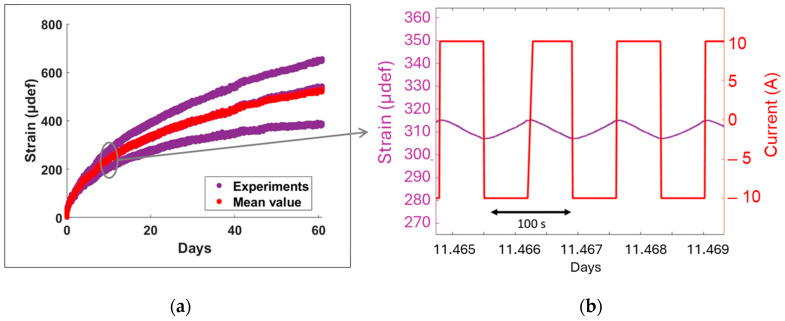
Raw strain results from strain gauges implemented on three supercapacitors: (**a**) strain over time over long duration; (**b**) strain and current evolution: zoom on a short duration.

The strain results from the 60 days of experiments on three identical SCs cycled at 10 A and 50 °C are shown in Figure 11a. As observed, the strain increased over time for all SCs, indicating their progressive mechanical deformation. The rate of increase was initially high and then gradually slowed down to reach a more stable state, closely matching the trend of capacitance loss. This correlation indicates that the initial strain rise is caused by rapid early degradation, during which gas generation is more pronounced, leading to greater internal pressure and casing deformation. As the degradation rate decreased over time, gas evolution diminished, resulting in the stabilization of the measured strain. The significant variation among the three samples may be attributed to differences in material properties or manufacturing inconsistencies. However, the average curve closely follows the trend of the individual SCs, suggesting a relatively consistent strain behaviour across all samples.

Figure 11b provides a detailed view of the resulting strain and applied current in a dynamic regime, i.e., over a short duration. It is particularly noteworthy that two distinct deformation regimes emerge. The first regime corresponds to a slow evolution (as shown in Figure 11a), which can be assimilated to a creep-like phenomenon in the mechanical response over a long timescale. This behaviour reflects a progressive and cumulative trend, characteristic of structural ageing. The second regime highlights a deformation synchronized with the applied current over a short time, as displayed in Figure 11b, but with a much lower strain amplitude (~10 µdef). This periodic response may be correlated with several underlying mechanisms:

• energy losses within the SC, potentially inducing a thermal dilation of the packaging [40].
• reversible electrochemical phenomena, which can also contribute to the observed deformation [41].

Overall, this measurement reveals the existence of two distinct signatures: one associated with structural ageing (slow and irreversible behaviour); and the other with operational conditions (reversible response of low amplitude) [40]. This distinction provides valuable insights for interpreting degradation mechanisms and enables real-time characterization of the health state of systems integrating SCs.

It is interesting to assess the correlation between the strain data to the capacitance measurements in order to identify the dominant degradation mechanism of the component under the ageing condition. Two methods have been developed to predict capacitance loss for SCs. The first approach is based on the Langmuir isotherms, which assume that the capacitance loss is mainly due to the gas adsorption during ageing, leading to a reduction in the electrolyte-electrode surface [8], a part of this gas will also increase the internal pressure inside the casing. The second model attributes capacitance loss to the growth of Solid Electrolyte Interface (SEI). Since we monitor the pressure induced deformation of the SC casing, the first method related to the adsorption-based model seems to be a better approach to assess the capacitance loss.

**Figure 12:**
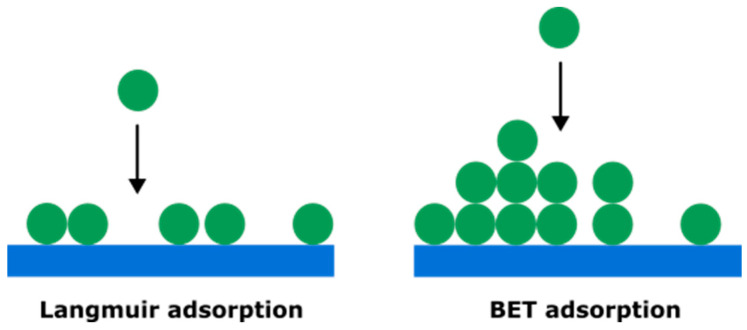
Langmuir model vs. BET model. The BET model, unlike Langmuir, allows particles to create infinite layers on top of previously adsorbed particles.

However, this approach can be improved. Equation (2) based on the Brunauer–Emmett–Teller (BET) model is revealed to be more suited to microporous materials than the Langmuir model [42,43]. In the Langmuir model, the adsorption of gas is considered to be a single layer, whereas the BET model allows an infinity of layers to be adsorbed at the electrode surface (see Figure 12).

In the BET, the adsorbed volume of gas [44] is given by:

(2) $$\frac{v}{V_{mono}}=\frac{\frac{P}{P_{sat}}}{\left(1\;-\;\frac{P}{P_{sat}}\right)\left(1\;-\;\frac{P}{P_{sat}}\;+\;c\;\times \;\frac{P}{P_{sat}}\right)}=\frac{x}{\left(1-\frac{x}{e}\right)\left(e+x\left(c-1\right)\right)}\;$$

where v and $V_{mono}$ are, respectively, the volume of adsorbed gas and of a single layer; P and $P_{sat\;}$ denotes the internal pressure of vapour and its saturation level; c is a constant and e is the vapour pressure; x denotes the strain that directly links to the pressure ratio:

(3) $$\;x=e\times \frac{P}{P_{sat}}$$

Equation (2) clearly shows a relationship between the pressure and the volume of the adsorbed gas. Cycling at 50 °C (and 60 °C) accelerates ageing and increases gas production, leading to increased strain.

Based on literature and post-mortem analyses, both the carbon electrode material and acetonitrile electrolyte are known to degrade under electrochemical stress, producing gaseous species such as carbon dioxide (CO_2_), carbon monoxide (CO), and hydrogen (H_2_) [21,45]. More explicitly, Pameté et al. proposed that the degradation of carbon electrodes in acetonitrile-based EDLCs involves simultaneous reduction and oxidation reactions [21]. At the negative electrode, carbon undergoes reduction, while at the positive electrode, oxygen-containing surface groups such as carbonyl and carboxyl are oxidized. Additionally, CO_2_ and H_2_O are reduced at the negative electrode, forming gaseous species like CO and H_2_, as well as carbonate ions (CO_3_^2−^). These gases accumulate continuously within the cell casing, leading to pressure buildup and mechanical deformation, which is captured by strain measurements.

**Figure 13:**
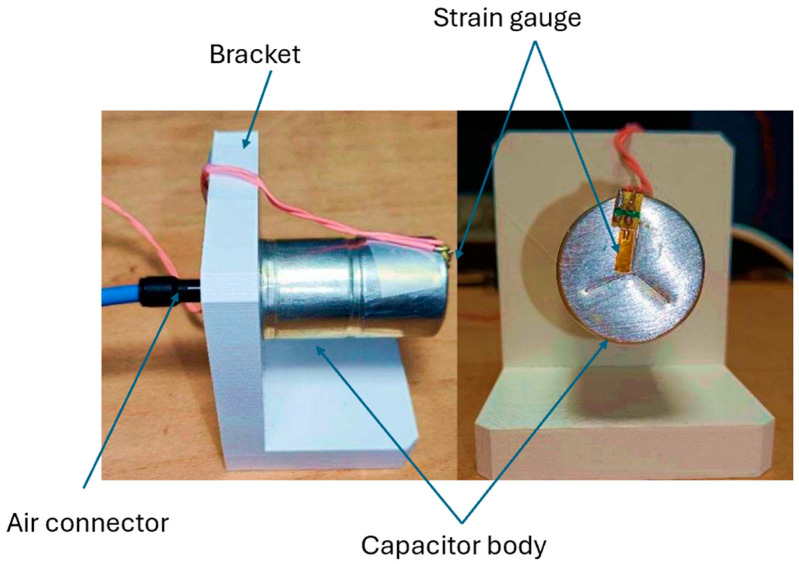
Experimental test bench for simulating the strain response of a casing as a function of air pressure.

To achieve the internal pressure measurement, the SC was opened and emptied, leaving only the casing that was then mounted onto a 3D-printed part. A strain gauge was installed on the top side of the casing, and pressure-strain data was collected using an electronic barometer (Figure 13). The protocol involved gradually increasing the pressure and making stops at various pressure levels to allow the casing to stabilize, ensuring that inertia was not considered.

Once the instrumentation was implemented, the measurements revealed the possibility of tracking different parameters related to charge–discharge cycles (Figure 2), as well as a “creep” phenomenon over longer timescales. These results may be influenced by several factors, such as the type of adhesive used for bonding the gauges, the sensor technology, or the compensations applied within the Wheatstone bridge configuration [26]. Nevertheless, precautions were taken, drawing on both prior experience and literature in related fields, such as civil engineering, where this type of sensor is routinely used for structural health monitoring (e.g., bridges) [46]. The observed fluctuations reflect mechanisms occurring within the structure itself, transmitted to external packaging, thereby highlighting the relevance of this approach for SC health monitoring.

To substantiate this hypothesis, a correlation was established with several relevant indicators including strain, capacitance, and as the ESR [47]. These analyses are expected to reinforce the validity of the proposed methodology.

**Figure 14:**
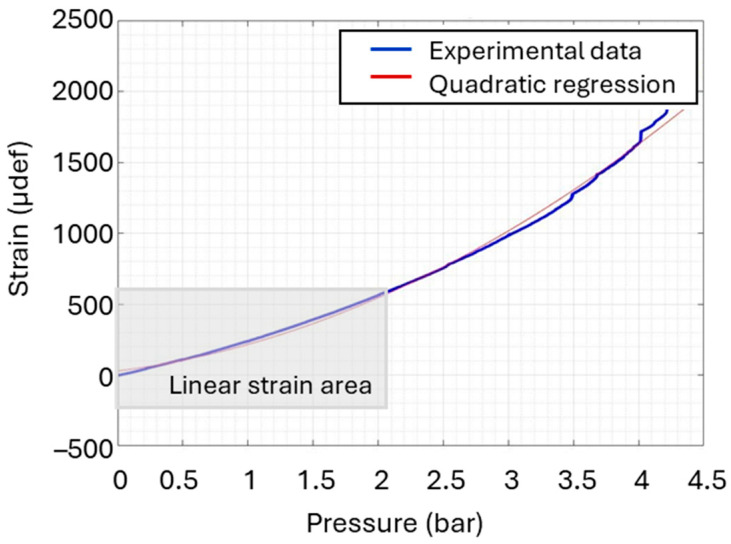
Pressure over strain fitted with a quadratic model.

As presented in Figure 14, the strain increases with pressure in a nonlinear manner and can be modelled as a second-order polynomial. The model closely follows the experimental data, reflecting a good correlation between pressure and strain. The quadratic behaviour in strain with pressure suggests a progressive softening effect of the material, meaning the structure deforms more easily as pressure increases. Interestingly, the strain increases gradually at low pressures, exhibiting an almost linear trend. Regarding the result of Figure 11, after an ageing experience of 60 days, the measured strain reaches approximately 600 µdef, which falls within the quasi-linear mechanical behaviour range illustrated in Figure 14. This study focuses mainly on this region, where the applied pressure was kept below 2.5 bar.

Between 3.5 and 4.5 bar, the strain response exhibits a nonlinear, stepwise increase, suggesting that the aluminum casing is no longer deforming purely elastically but has entered a regime of plastic deformation. For aluminum, the upper limit of the linear elastic domain typically occurs around 1000 µstrain, corresponding to an offset yield strength of approximately 100 MPa [48]. The offset yield strength represents an approximate measure of the material’s elastic limit, defined as the stress corresponding to the intersection between the stress–strain curve and a line parallel to the initial elastic modulus.

**Figure 15:**
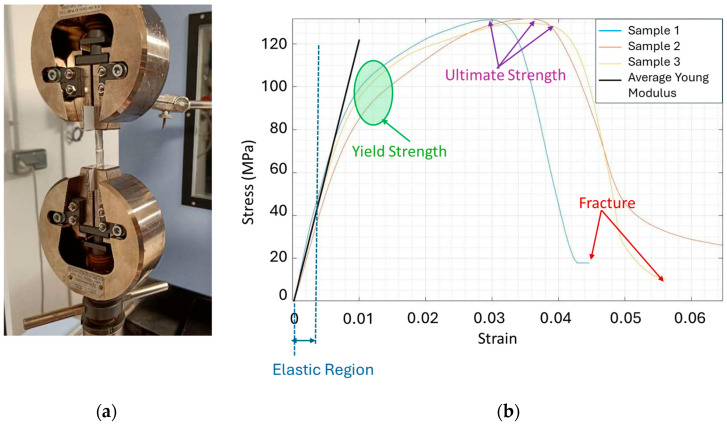
(**a**) Tensile mechanical setup; (**b**) Stress-versus-strain behaviour.

To confirm this behaviour, tensile tests were conducted in three supercapacitor casings, as shown in Figure 15a. Their corresponding stress–strain response, displayed in Figure 15b, are consistent with the typical behaviour of aluminum alloys. The close similarity among the tested samples reflects the good reproducibility of the measurements.

**Figure 16:**
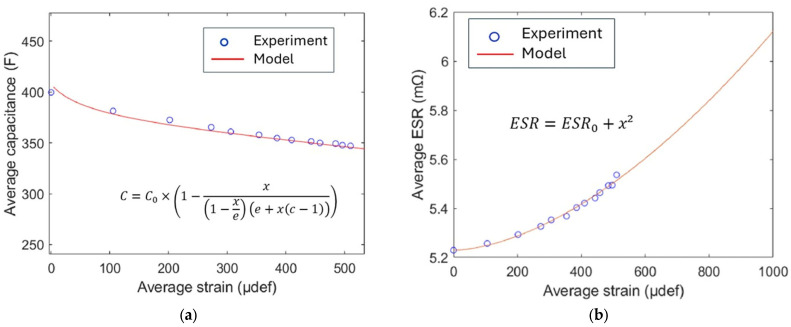
(**a**) Average capacitance, and (**b**) average resistance versus average strain.

From the measured pressure we can determine the corresponding strain and, ultimately, drive the model for the capacitance loss using the least-squares fitting method, as shown in Figure 16a. The capacitance decreases with strain, which could be attributed to material ageing and/or structural degradation. The shape of curve then resembles a type II adsorption isotherm, which is described by the BET theory.

For the ESR versus strain, a second-order polynomial function has been chosen empirically, as displayed in Figure 16b. The coefficients were then determined using the least squares fitting method. The nonlinear behaviour between *ESR* the strain can be interpreted based on several complementary hypotheses.

First, the role of casing, which, due to its nature and integration, acts as a thermal dissipator, thereby establishing a correlation between ESR evolution and Joule heating losses. This thermal effect may be coupled with internal degassing phenomenon, as discussed in the literature [34,49], which an further contribute to the observed nonlinear effects.

Second, within the strain range of around 500–600 µstrain, the casing material approaches a nonlinear mechanical regime (cf. Figure 14), as confirmed by the mechanical tests showing that strain no longer evolves proportionally with the applied stress.

Third, geometric factors may also play a role. The mechanical configuration of the instrumented zone can be approximated as a diaphragm-like structure, where bending behaviour becomes nonlinear beyond a certain deflection threshold [50,51].

In summary, the possible causes of a nonlinear response may be attributed either to the intrinsic behaviour of the materials composing the supercapacitor packaging, or to extrinsic effects such as thermally induced phenomena arising from losses, as well as gas release due to the ageing of the overall structure.

Moreover, the EoL for SCs is defined by the ISO 12405-4:2018 [52], which specifies a 20% decrease in capacitance and a 200% increase in ESR.

The decline in SC function is mainly attributed to the fading of capacitance during ageing. Thus, the State of Health corresponding to such a capacitance change (denoted as SoH_c_) can be defined by the following equation:

(4) $$\;SoH_{c}=\frac{C_{act}}{C_{init}}$$

with $C_{act}$ is the current capacitance and $C_{init}$ the initial capacitance.

This definition does not account for the resistance change. Therefore, it can be useful to express the SoH through the remaining energy of the SC (denoted as SoH*_e_*), which provides an estimate of the remaining useful cell life during ageing. Regarding the charging step of the capacitor within a voltage range of 0–2.7 V, the SoH*_e_* indicator can be calculated as:

(5) $$\;SoH_{e}=\frac{\frac{1}{2}C_{act}V_{r}^{2}-\;ESR_{act}I^{2}\Delta t}{\frac{1}{2}C_{init}V_{r}^{2}-\;ESR_{init}I^{2}\Delta t}$$

where Δt, which presents the time needed to raise the voltage from 0 to 2.7 V (V_r_), is expressed based on the standard capacitor equation:

(6) $$\;\Delta t=\frac{C\times V_{r}}{I}$$

Since ΔU corresponds to the full voltage range during the characterization phase, the final expression is given by:

(7) $$\;SoH_{e}=\frac{\frac{1}{2}C_{act}V_{r}^{2}-\;ESR_{act}I\times C_{act}V_{r}}{\frac{1}{2}C_{init}V_{r}^{2}-\;ESR_{init}I\times C_{init}V_{r}}$$

**Figure 17:**
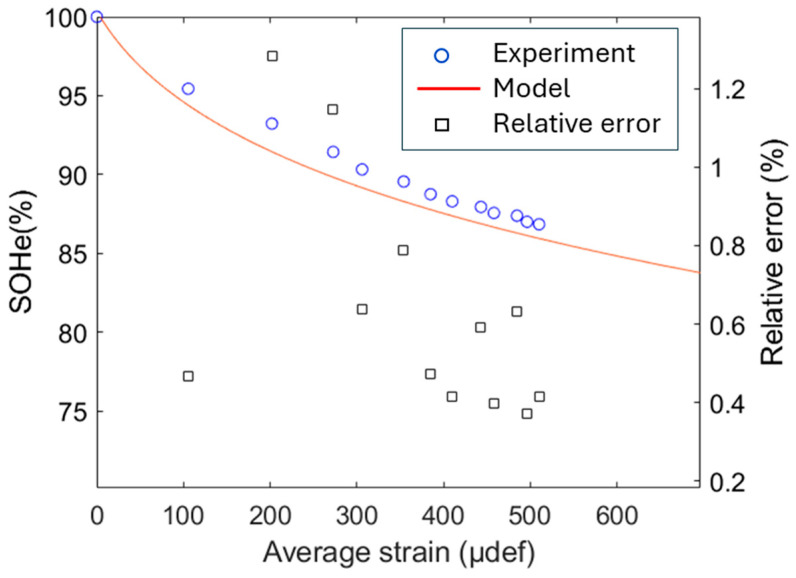
Experimental and model data of SoH_e_.

Combining the two models of ESR and capacitance decrease, it becomes possible to predict the state of health through the remaining energy inside the cell (SoH*_e_*), as illustrated in Figure 16. SoH*_e_* gradually declines from 100% (initially under free strain) to ~80% (at 1000 µdef). The close alignment between the analytical model (blue line) and empirical data (cyan markers) confirms that the model provides a reliable prediction of SOHₑ under varying strain conditions. The relative error (red squares in Figure 17) is defined as follows:

(8) $$\;Relative\;gap=\left|\frac{SoH_{e|experiment}-SoH_{e|model}}{SoH_{e|experiment}}\right|$$

It mostly remains between 0.4% and 1.2%, indicating a somewhat small deviation between the model and real data, supporting the model’s accuracy.

Based on the above analysis, strain measurement appears to be an effective indicator for monitoring the state of health of SCs. The strong correlation between strain and relevant physical parameters such as the remaining energy and capacitance change, along with the close alignment between the model and experimental data, confirms that strain can serve as a reliable diagnostic parameter for assessing SC ageing and performance degradation.

Moreover, it is important to highlight that most diagnostics currently performed on energy storage systems rely on invasive approaches or require measurements that necessitate shutting down the system in order to characterize its electrical properties. As emphasized in the Introduction, these approaches are limited by technological and operational constraints, and are often impractical in applications such as aeronautics or heavy industry, where reliability and continuity of operation are critical [15,16,17,53]. The present study therefore aims to identify a non-invasive ageing indicator for supercapacitor-based storage systems. While invasive methods remain more precise for identifying detailed ageing mechanisms, the proposed approach provides a valuable step toward continuous, in situ monitoring of device health.

## 4. Conclusions

This work provides an assessment of the ageing characteristics of commercial supercapacitors, while also demonstrating the effectiveness of mechanical deformation analysis as a method for monitoring their SoH. A detailed analysis of the ageing behaviour—specifically, capacitance decrease and resistance increase—was conducted.

Gas emission inside the cell is a primary ageing mechanism that can be monitored through casing deformation caused by increased internal pressure. Strain gauges provide a non-invasive method for measuring this deformation effectively. Finally, a direct correlation between capacitance fading, resistance increase, and the SoH of the cell was established. Strain gauges demonstrate significant potential as a non-invasive technology for monitoring the SoH of supercapacitors.

Future research could explore the use of rosette strain gauges to enhance measurement accuracy, as well as flex-PCB sensors for improved integration. Additionally, extending the experiments to a broader range of temperatures would enable a systematic evaluation of the temperature dependence of the identified ageing indicators. Such studies could provide a more comprehensive understanding of the degradation mechanisms and allow for a more robust assessment of the indicators under varying thermal conditions.

## Figures and Tables

**Table 1:** Specifications of the investigated supercapacitors [33].

**Eaton XV3560-2R7407-R**	
Nominal Capacitance	400 F
Rated Voltage (V_r_)	2.7 V
Maximum continuous current	26 A
Peak current	220 A
Operating temperature	−40 °C to +65 °C
Initial ESR	3.2 mΩ
Stored energy (E)	0.41 Wh
Max power	570 W
Length	63 mm
Diameter	35 mm
Typical mass	72 g
Cycle life ^1^	500,000

^1^ Cycled at room temperature between max operating voltage and 50% of max operating voltage.

## Data Availability

The original contributions presented in this study are included in the article. Further inquiries can be directed to the corresponding author.
